# The complex role of kininogens in hereditary angioedema

**DOI:** 10.3389/falgy.2022.952753

**Published:** 2022-08-03

**Authors:** Allen P. Kaplan, Kusumam Joseph, Berhane Ghebrehiwet

**Affiliations:** ^1^Medicine/Pulmonary and Critical Care, Medical University of South Carolina, Charleston, SC, United States; ^2^BioCryst Pharmaceuticals, Durham, NC, United States; ^3^Department of Medicine, Stony Brook University, Stony Brook, NY, United States

**Keywords:** angioedema, bradykinin, kininogen, kallikrein, vascular permeability

## Abstract

Human high molecular weight kininogen (HK) is the substrate from which bradykinin is released as a result of activation of the plasma “contact” system, a cascade that includes the intrinsic coagulation pathway, and a fibrinolytic pathway leading to the conversion of plasminogen to plasmin. Its distinction from low molecular weight kininogen (LK) was first made clear in studies of bovine plasma. While early studies did suggest two kininogens in human plasma also, their distinction became clear when plasma deficient in HK or both HK and LK were discovered. The light chain of HK is distinct and has the site of interaction with negatively charged surfaces (domain 5) plus a 6th domain that binds either prekallikrein or factor XI. HK is a cofactor for multiple enzymatic reactions that relate to the light chain binding properties. It augments the rate of conversion of prekallikrein to kallikrein and is essential for the activation of factor XI. It indirectly augments the “feedback” activation of factor XII by plasma kallikrein. Thus, HK deficiency has abnormalities of intrinsic coagulation and fibrinolysis akin to that of factor XII deficiency in addition to the inability to produce bradykinin by factor XII-dependent reactions. The contact cascade binds to vascular endothelial cells and HK is a critical binding factor with binding sites within domains 3 and 5. Prekallikrein (or factor XI) is attached to HK and is brought to the surface. The endothelial cell also secretes proteins that interact with the HK-prekallikrein complex resulting in kallikrein formation. These have been identified to be heat shock protein 90 (HSP 90) and prolylcarboxypeptidase. Cell release of urokinase plasminogen activator stimulates fibrinolysis. There are now 6 types of HAE with normal C1 inhibitors. One of them has a mutated kininogen but the mechanism for overproduction (presumed) of bradykinin has not yet been determined. A second has a mutation involving sulfation of proteoglycans which may lead to augmented bradykinin formation employing the cell surface reactions noted above.

## Introduction

When one considers the plasma pathway(s) for the production of bradykinin, a role for kininogen was determined many years before any role for factor XII or prekallikrein was discerned and bovine proteins were purified and at least partially characterized before the corresponding human proteins were identified. Defined as substrates from which bradykinin is derived, the role of the various kininogens in actually producing bradykinin *in vivo*, is far more complex. This review article is based on a lecture given at the Hereditary Angioedema Conference in Budapest, Hungary in 2020, and includes updates representing the most recent discoveries since then.

## The earliest history: How many kininogens are there?

While it may seem obvious to any worker in the bradykinin “field” or those involved with the pathogenesis and/or treatment of hereditary angioedema (i.e., C1 inhibitor deficiency) that the answer is two kininogens, this was not always clear, and if one studies rodent kininogens, particularly in the rat, the correct answer is three! Nevertheless, workers in Japan purified two kininogens from bovine plasma and did find them to differ in molecular weight, hence the names low molecular weight kininogen (LK) and high molecular weight kininogen (HK) (1, 2). Those working with human plasma questioned whether the smaller form is a degradation product of the larger or perhaps the larger represents an aggregate of the smaller, based in part because early purifications suggest just one species (3). Other workers disagreed, and felt that two forms were more likely based on two observations, namely, (A) two kininogens of differing size were identified upon fractionation of human plasma and (B) partially purified kininogen preparations showed different kinetics i.e., different values for *K*_m_, *V*_max_, and/or *K*_cat_ when digested either by a plasma kallikrein preparation or tissue kallikrein (4, 5). The larger form seemed to be a preferential substrate of plasma kallikrein (6).

## The answer emerges: There are two human kininogens and one of them is a clotting factor

Experiments of nature came to the rescue with the discovery of three plasmas that were deficient in one or both kininogens and were named for the patients affected; namely, Fitzgerald, Williams, and Flaujeac (7–9). These plasmas failed to produce bradykinin when “activated” by a negatively charged surface, e.g., kaolin, which was routinely used in the 1960's and 1970's. However, the partial thromboplastin time (PTT) was as abnormal as factor XII deficiency (8) and factor XII-dependent fibrinolysis was also markedly prolonged. The patients were generally well and Mrs. Williams, in particular, was identified when her pre-operative laboratory values were assessed prior to an elective cholecystectomy and her plasma failed to clot. William's plasma turned out to have a deficiency of all kininogens, both LK and HK (8) while Flaujeac plasma had a selective deficiency of HK (9). Subsequently, the defect in Fitzgerald plasma was confirmed to be due to HK deficiency. Our studies of the Williams' plasma are summarized below.

It was quickly determined that Williams plasma lacked kininogen. At that time (1975), we had factor XII fragment (β-FXIIa) and preparations of active kallikrein. Incubation of William's plasma with these enzymes produced no bradykinin. We had no source of purified kininogen, however, heating normal plasma to 61 °C for 2 h. destroyed all enzymes needed to produce bradykinin, kininogen was still viable, so that incubation with plasma kallikrein produced bradykinin while incubation with factor XIIf did not (prekallikrein had been destroyed). Thus, the addition of heated plasma to Williams plasma and the addition of either factor XIIf or kallikrein not only produced bradykinin but also corrected the coagulation defect (abnormal PTT. By that time, (1975), Jack Pierce had preparations of kininogens that differed in size and susceptibility to plasma kallikrein (the larger ones) or tissue kallikrein (the smaller ones) except for one, which was small but was mainly cleaved by plasma kallikrein (5, 8). When samples of each were numbered and studied blindly, all the larger ones corrected the William's plasma defects, but the small ones did not, except for the one “small” preparation that was susceptible to plasma kallikrein (8). His chromatographic separations were so sensitive, that he had numbered 15 kininogens that sorted into these two main groups, and later once we purified LK and HK, we realized that he was discerning the carbohydrate heterogeneity of these glycoproteins. The one small protein that functioned as HK was probably an HK cleavage product that may actually exist in plasma (10).

**Figure 1:**
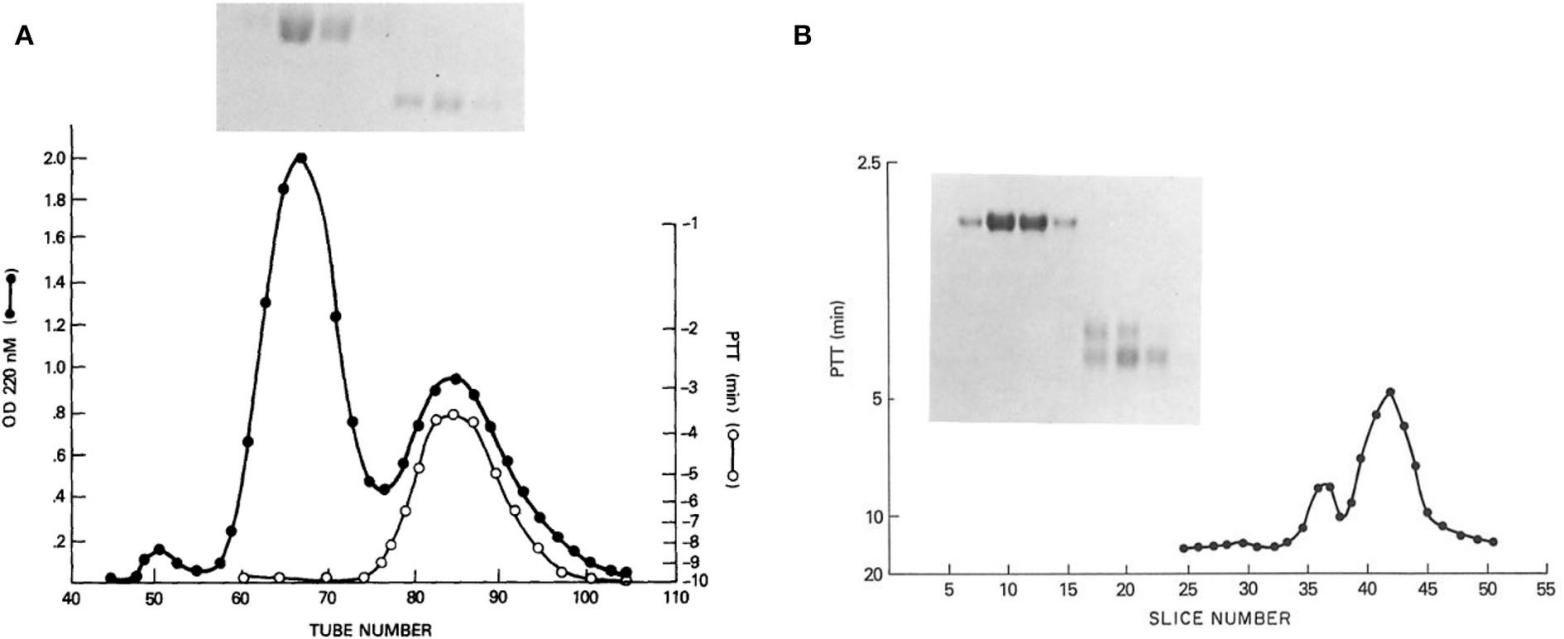
**(A)** Sephadex G-200 gel filtration of reduced kinin-free HMW kininogen. The absorbance at 280 nm is shown (black line) in addition to the ability of fractions to correct the partial thromboplastin time of HK kininogen-deficient plasma (white line). Above (inset) is shown the SDS-PAGE pattern obtained after electrophoresis of 100 μl of tubes 60, 65, 70, and 75 (heavy chain), and electrophoresis of 100 μl of a 10-fold concentration of tubes 80, 85, and 90 (light chain). **(B)** Urea disc gel electrophoresis of samples taken from the Sephadex G-200 gel filtration of reduced kinin-free HWM kininogen [in this figure **(A)**]. A 100 μl of tubes 60, 65, 70, and 75 followed by 100 μl of a 20-fold concentration of tubes 80, 85, and 90 were applied. A replicate gel was sliced, each slice was eluted with 0.2 ml phosphate-buffered saline, and the eluates were assayed for their ability to correct the partial thromboplastin time of HMW kininogen-deficient plasma. The peaks of coagulant activity seen at slices 36–38 and 40–44 correspond to the two fainter light chain bands seen on the right side of the gel.

From these results, suggesting two kininogens, HK and LK, with all the functions associated with HK, we proceeded to purify kininogens from 10 L of human plasma, facilitated by the identification of HK by correction of the PTT of Williams plasma and an anti-kininogen antibody reactive with any kininogen (11). The isolated proteins had single bands on disc gel electrophoresis. The HK preparation was cleaved with plasma kallikrein and the kinin-free protein was reduced and alkylated and fractionated by Sephadex G200. Peaks identified as heavy chain and light chain (Figure 1A) were assayed for the ability to correct the PTT of Williams plasma and all the activity was in the light chain (8). Similarly, the fibrinolytic defect was also reversed by the addition of a light chain but not a heavy chain. While the molecular weights calculated were 66,000 and 37,000 respectively, urea-disc gel electrophoresis revealed light chain “activity” at 56,000 and 37,000 and the larger was converted to the smaller representing an additional cleavage of the light chain without loss of function (Figure 1B).

## Assembly of bradykinin-forming proteins in plasma

During the course of these studies, prekallikrein was purified (12) and was found to have a molecular weight of 80,000. Conversion to kallikrein requires a single cleavage within a disulfide bridge at an Arg-Ile bond so that the active enzyme has a heavy chain disulfide-linked to a light chain (actually two forms of the light chain because of carbohydrate heterogeneity) but no change in total molecular weight, i.e., 80,000 Kd. Earlier, there was a publication by Nagasewa et al. who fractionated whole plasma and determined the molecular weight of prekallikrein to be 280,000 (13). We surmised that this difference in size could be due to the binding of a protein in plasma, and HK seemed to be a reasonable candidate. We determined a molecular weight of 285,000 for prekallikrein in normal plasma (14), and next fractionated Williams plasma on Sephadex G200. The value was 120,000 (typical of gel filtration −80,000 on SDS gel electrophoresis). We concluded that prekallikrein circulates bound to HK. We then used I^125^ labeled prekallikrein to prove the point. When added to Williams plasma, the radioactivity traveled with a molecular weight of 120,000. When Williams plasma was reconstituted with purified HK, ^125^I prekallikrein was added, and the plasma was then subjected to gel filtration, the ^125^I prekallikrein was then found at 300,000 indicating interaction with HK to form a complex (14). The percentage of binding depends on the concentration of the reactants and the binding constant. Subsequent analysis revealed that about 75% of prekallikrein is bound, so up to 25% can be present free (15). We assessed the molecular weight of factor XII throughout these experiments and found it to be the same in plasma as that of the purified protein, and concluded that it circulates unbound to other plasma constituents. Given the findings that the light chain derived from kinin-free HK possesses all of the clotting and fibrinolytic potential of HK, we compared the binding of prekallikrein to the purified heavy and light chains, and binding to the light chain but not the heavy chain was affirmed (16).

**Figure 2:**
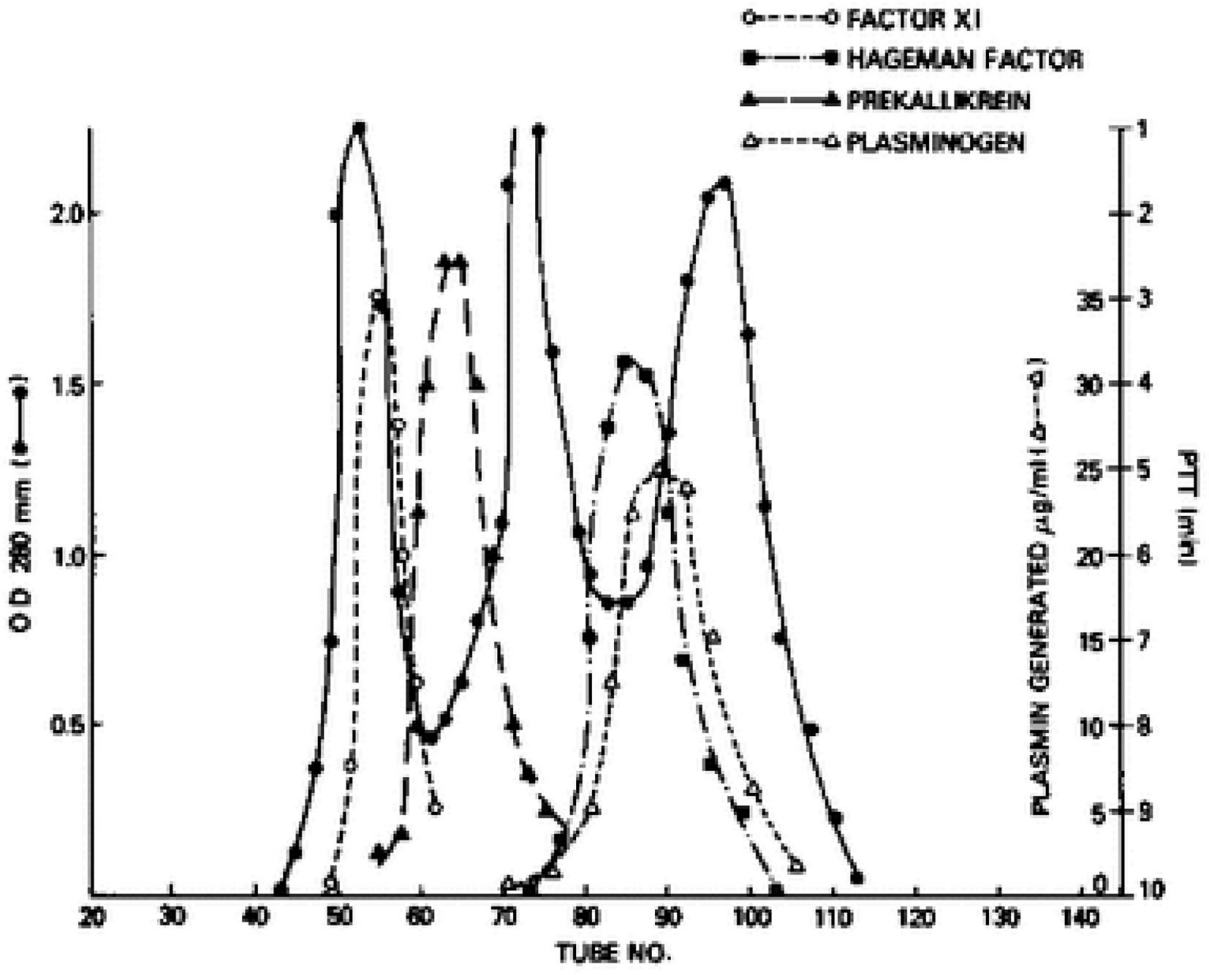
Gel filtration of human plasma on Sephadex G-200. The OD 280 indicates 3 protein peaks. The location of factor XI, prekallikrein, factor XII (Hageman factor) and plasminogen was identified immunologically. The molecular sizes of factor XI and prekallikrein reflect the binding of each to HK.

We next turned our attention to factor XI, even though it had a known role in the generation of bradykinin. Factor XI has four tandem repeats of about 90 amino acids each along the N-terminus of the protein that are homologous to repeats that is only in prekallikrein (17). Thus, they are more closely related to each other structurally than any other plasma protein. So, we repeated the aforementioned experiments with factor XI in place of prekallikrein. Factor XI is dimeric (18, 19) i.e., two 80,000 Kd subunits are disulfide-linked, so its molecular weight purified, or in Williams plasma is 160,000. However, its molecular weight in normal plasma is over 400,000 (20), and we could observe the increase in the apparent size of ^125^I-factor XI when added to Williams plasma reconstituted with HK by gel filtration. Again, binding was to HK-derived light chain and not a heavy chain (16, 20). Although the plasma concentration of factor XI is less than prekallikrein (7 μg/ml vs. 25 μg/ml), about 99% of factor XI circulates bound to HK indicating a more avid attachment. Further, the dimeric structure of factor XII indicates that it is possible for one molecule of factor XI to bind two molecules of HK, one for each subunit. Figure 2 is a fractionation of normal plasma depicting the factor XI-HK complex as the largest, followed by the prekallikrein HK complex. The second protein peak is IgG, which marks 160,000. The factor XII and plasminogen peaks are shown along the ascending limb of the third protein peak, albumin, at 60,000.

The amino acid sequences required for binding of prekallikrein and factor XI are not identical although there is some overlap. Factor XI binds to residues 185–242 of the light chain of HK (21) and prekallikrein binds to residues 185–224 (22). There is no effective competition for binding within plasma because the HK concentration at 80 μg/ml, when converted to moles present, is sufficient (theoretically) to bind virtually all the factor XI and prekallikrein present.

## The domain structure of the HK proteins

**Figure 3:**
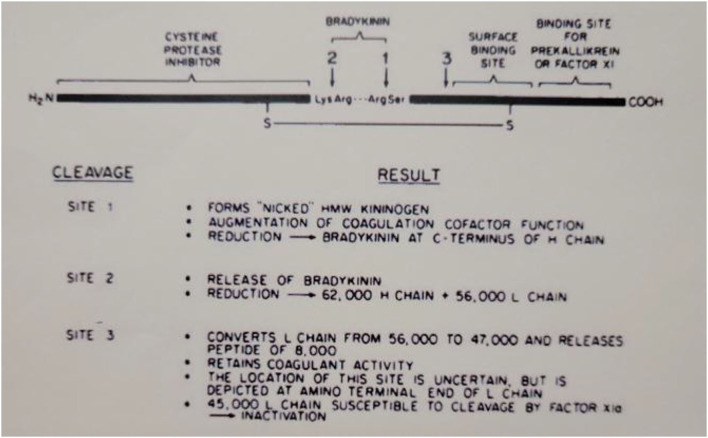
Depiction of the structure of human HWM kininogen and the consequences of cleavages at the sites indicated. The amino-terminal portion (heavy chain of cleaved kininogen) has the cysteine protease inhibitor activation. The light chain of cleaved HWM kininogen serves as the cofactor function in coagulation, fibrinolysis, and initiation of bradykinin formation.

The domain structure of HK is quite complex and clearly assembled from exons that may have evolved from smaller proteins with very varied functions. The N-terminal 4 domains are shared with LK and encompass the heavy chain (domains 1–3) and the bradykinin sequence plus an additional 10 amino acids (domain 4). The first 3 domains are homologous to each other and are derived from smaller entities known as cystatins (23) whose ancestors are still smaller proteins known as stefins (24). The cystatins are cysteine protease inhibitors, e.g., they inhibit papain or cathepsins B, H, and L (25). Domains 2 and 3 of the heavy chain both retain this enzyme inhibitory activity. Domain 1, while still homologous, lacks inhibitory function. This is depicted in Figure 3, which includes all six HK domains, with domains 1–3 indicated by “cysteine protease inhibitor.” Domain 4 includes the bradykinin amino acid sequence plus 10 amino acids. This is the point of divergence of HK and LK, so they are exactly alike for domains 1–4. Domains 5 and 6 are unique to HK and are responsible for all of its functions in “contact activation,” i.e., the plasma bradykinin-forming cascade. Domain 5, also known as the histidine-rich region, is the main site for interaction of HK with negatively charged substances/surfaces when used to activate the cascade *in vitro* such as kaolin, dextran sulfate, or ellagic acid. Domain 6 is critical and has the site(s) for interaction with prekallikrein and factor XI. The entire amino acid sequence analysis and domain structure for high and low molecular weight kininogens have been published (26, 27). One of the main *in vivo* binding interactions of HK is with vascular endothelial cells (discussed below), and here there are binding sites both in domain 3 in addition to domain 5 (28, 29).

When bradykinin-free HK is reduced and alkylated, the heavy chain contains domains 1–3 and the light chain has domains 5 and 6 plus the 10 C-terminal amino acids of domain 4. Thus, the light chain possesses all of the cofactor functions of HK, i.e., augmentation of the rate of various enzymatic reactions (30) as discussed below.

## HK is a co-factor for intrinsic coagulation, fibrinolysis, and bradykinin formation

While plasma lacking HK cannot produce bradykinin by activation of factor XII (i.e., there is no substrate for plasma kallikrein), it becomes evident why coagulation and fibrinolysis are about as abnormal as they are in factor XII-deficient plasma. Four issues need to be considered (A) HIK augments the rate of conversion of prekallikrein to kallikrein (30). Since the kallikrein-feedback activation of factor XII is rate limiting for factor XII activation (31, 32), there is a slower rate of factor XII activation. (B) When negatively charged surfaces (kaolin, dextran sulfate) initiate the cascade, factor XI activation is totally dependent on attachment to the surface *via* HK; it is not activated in the fluid phase. Thus, factor XI is not activated and intrinsic coagulation does not proceed. (C) Intrinsic fibrinolysis is complex and dependent on multiple enzymes to convert plasminogen to plasmin. These include factor XIIa (33), plasma kallikrein (34), and factor Xia (35) plus kallikrein activation of the small amount of prourokinase in plasma to urokinase (36). Activation of all these enzymes, including prourokinase is retarded by the above consequences of HK deficiency. (D) The fourth consideration is more subtle. It is evident that the kallikrein feedback is impaired if kallikrein formation is slowed by HK deficiency. However, it has been shown that the cleavage of surface-bound factor XII by kallikrein (already formed) is augmented upon the addition of HK (31). The most likely explanation is that kallikrein, in the absence of HK, binds firmly to the negatively charged surface, thus limiting its ability to activate factor XII. However, binding the HK-prekallikrein complex occurs *via* HK, so that some of the kallikreins thus formed can dissociate from HK and as it dissociates can pass along the cell surface to activate multiple factor XII molecules (37, 38).

**Figure 4:**
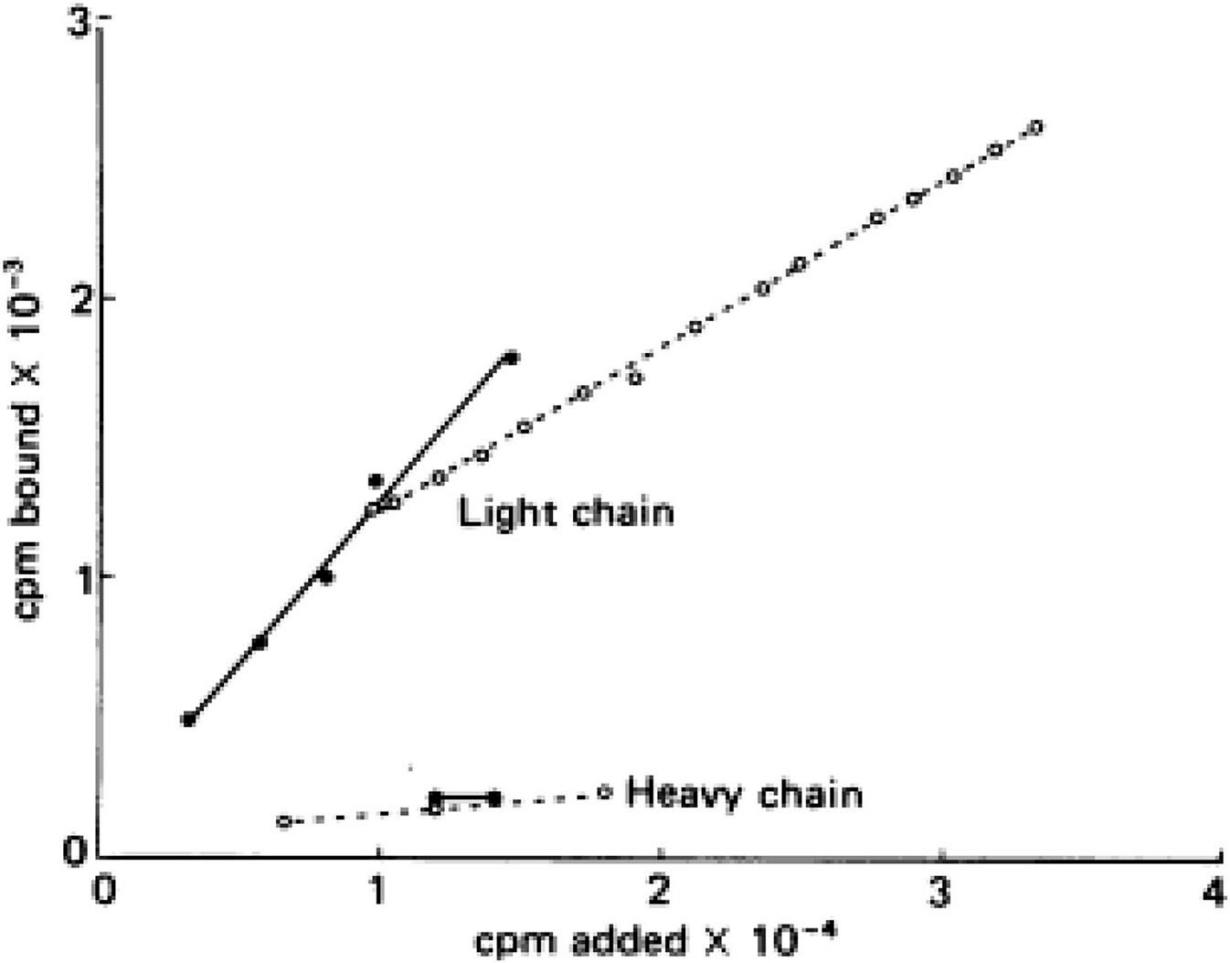
Addition of ^125^I—factor XI (solid lines) or ^125^I—prekallikrein (dotted line) to surface-bound light chain or heavy chain purified from HK. The counts bound (vertical) per moles added (horizontal) are plotted.

**Figure 5:**
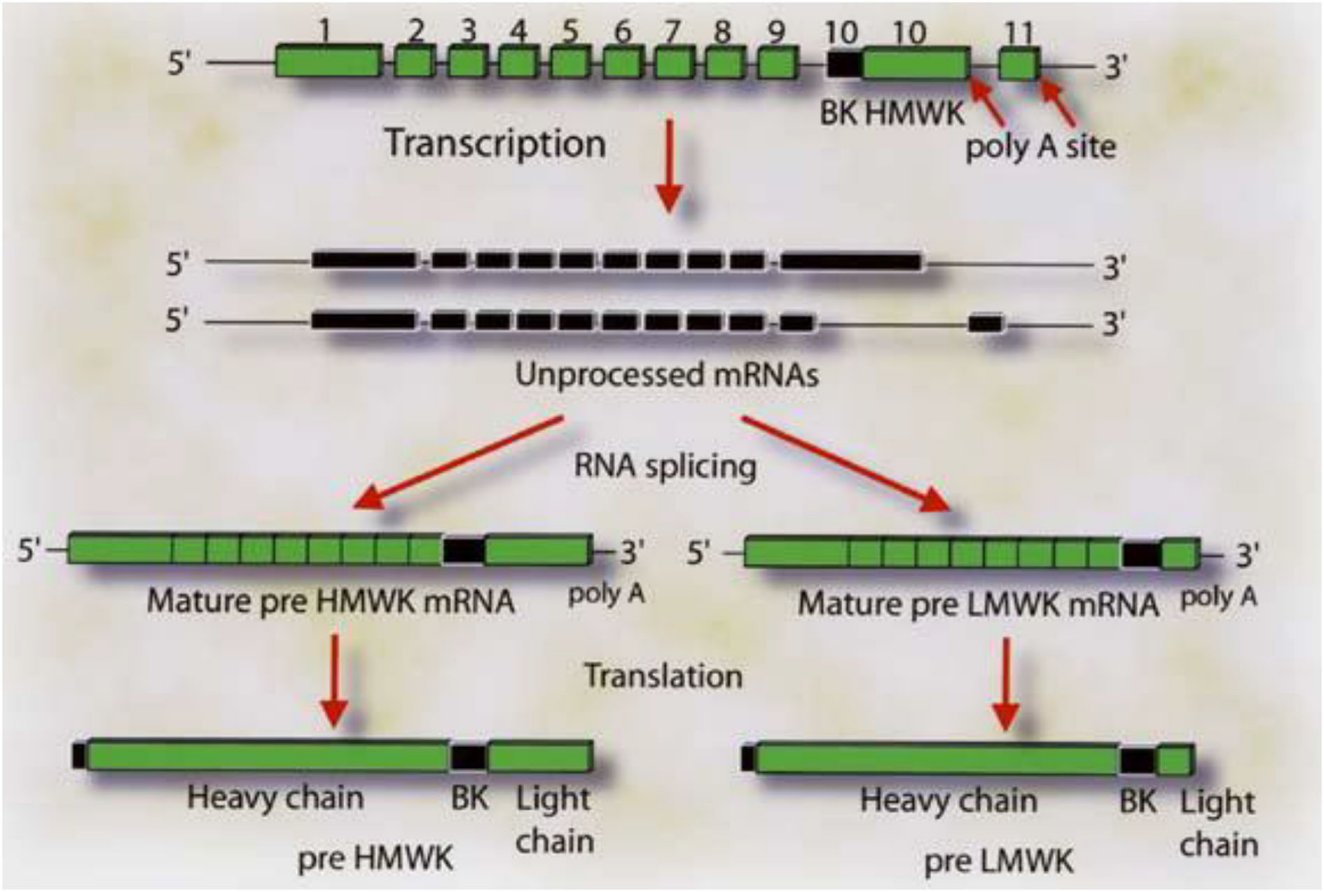
The gene structure for HK and LK. The boxes labeled 1–9 represent the exon coding for the heavy chain of HK and LK. Exon 10 codes for the bradykinin sequence and the light chain of HK. The respective mRNA's are assembled by alternative spicing events in which the light chain sequences are attached to the 3′ end of the 10 amino acid sequence C-terminal to bradykinin. Attachment of exon 10 in its entirety produces HK. Splicing of exon 10 (blackened portion) to exon 11 produces LK. Reproduced from Kitamura et al. (27).

Virtually all the above reactions, except bradykinin formation, are corrected with purified light chains derived from HK, while LK has no activity. An example of such light chain function is shown in Figure 4 which depicts the binding of ^125^I light chain to prekallikrein and factor XI with ^125^I heavy chain as the negative control. The exon/intron structures of HK and LK (27) are shown and contrasted in Figure 5. Multiple exons are typically linked to produce one domain. The critical point is that the mechanism of alternative splicing accounts for the difference in the C-terminal portion (or light chains) of HK and LK. The splice site is within exon 10. Then either exon 11 is attached to produce the small light chain of LK, or the entire exon 10 is attached to produce the light chain of HK, and accounting for domains 5 and 6 of HK.

## Interaction of HK with endothelial cells

**Figure 6:**
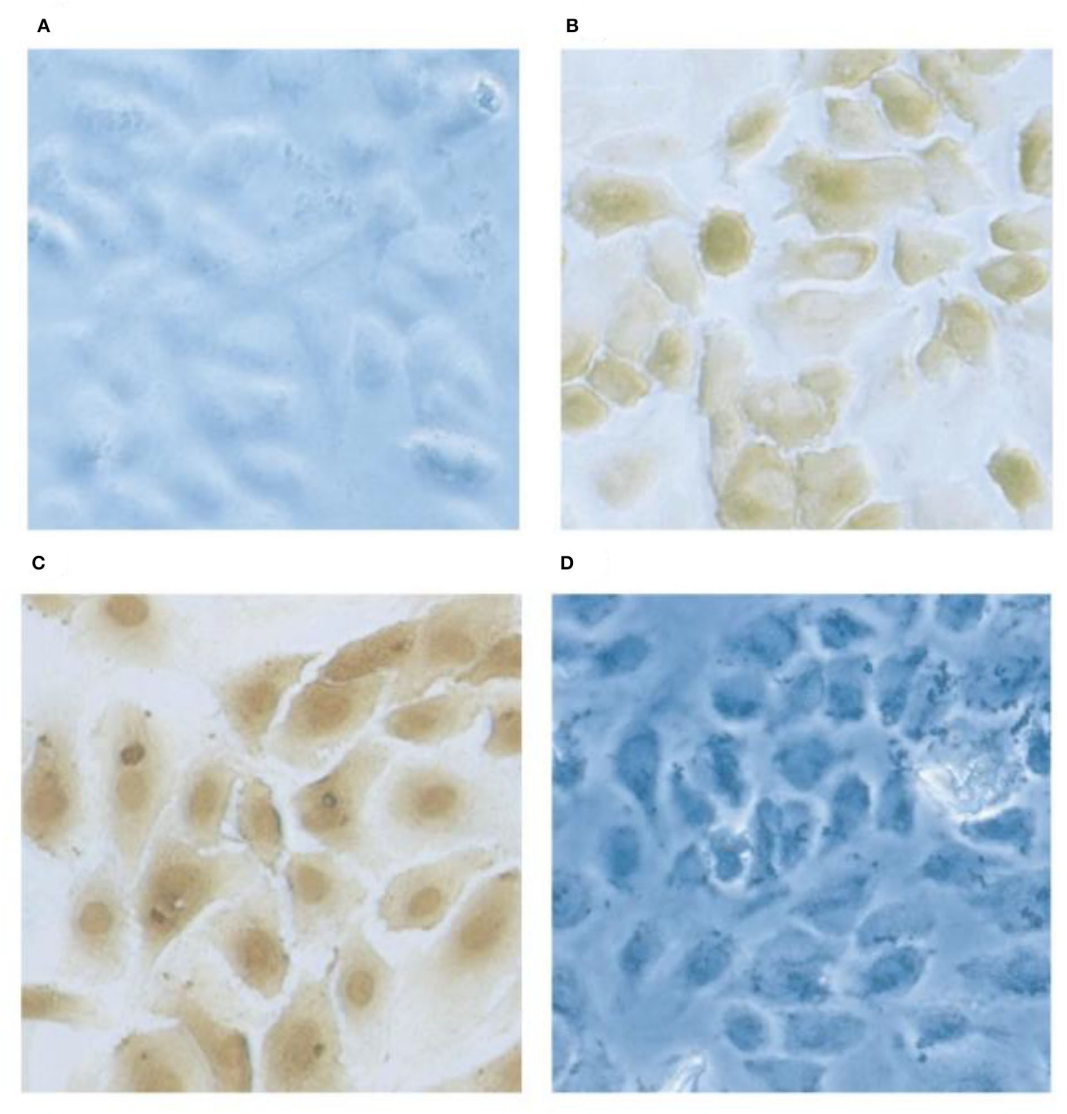
Localization of gC1qR on HUVEC's by immunochemical staining. Monolayer cultures of HUVECs on slides are fixed using 4% formaldehyde. The cells were first probed with rabbit anti gC1qR antibody and subsequently with horse radish -labeled goat anti-rabbit IgG. The gC1qR was visualized by 3,3-diaminobenzidine. Preimmune rabbit IgG staining showed no signal **(A)** and the anti-gC1qR antibody stained at the cell surface in non-permobilized cells **(B)** while in permimobilized cells **(C)**, the perinuclear and nuclear regions were prominently stained. In **D**, the cells were treated as in **(B)** but the antibody was pretreated with excess recombinant gC1qR.

Factor XII and HK each bind to the surface of endothelial cells where activation of the various cascades can take place. Three binding proteins have been described (gC1qR, cytokeratin 1, and u-PAR-(urokinase plasminogen activation receptor) (39–41) which are assembled as bimolecular complexes consisting of gC1qR-cytokeratin 1 and cytokeratin 1-u-PAR (42). When binding to an individual protein is studied, HK binds to gC1qR via domain 5 (39) (and as such competes for the binding site with factor XII) but, HK binds to cytokeratin 1 by domain 3 (43) thus both domains 3 and 5 may be involved upon interaction with the gC1qR-cytokeratin 1 complex. The staining of human umbilical cord vascular endothelial cells (HUVEC) for the presence of surface gC1qR is shown in Figure 6. Although HK binds to u-PAR, the interaction is relatively weak and an HK affinity column failed to bind u-PAR from solubilized endothelial cell membranes from which gC1qR and cytokeratin 1 were readily isolated (43). Binding to the cell surface can be inhibited with a combination of antibodies directed to gC1qR plus cytokeratin 1 and in our hands, this can account for about 85% of total binding (43). The urokinase plasminogen activator receptor (along with gC1qR) is a major binding protein for factor XII (44).

**Figure 7:**
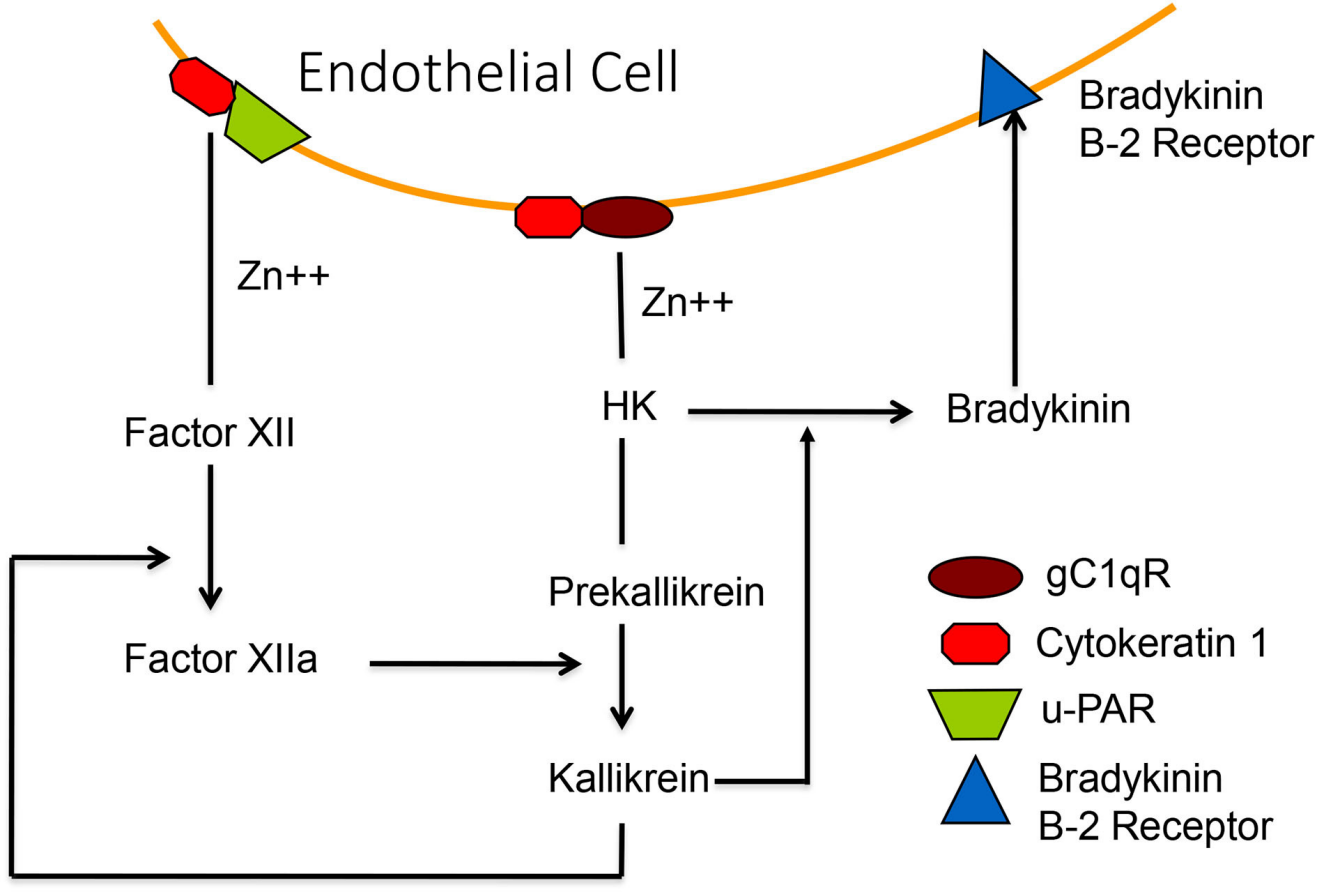
A diagrammatic representation of the zinc-dependent binding of HK-prekallikrein and factor XII to the surface, employing primarily complexes of gC1qR-cytokeratin-1 and cytokeratin 1—uPAR, respectively. Activation of factor XII, or activation of HK-prekallikrein by HSP 90, results in the formation of both factor XIIa and kallikrein. The latter digests HK to release bradykinin which binds to the B-2 receptor to increase vascular permeability.

The crystal structure for binding factor XII and/or HK to a trimer of gC1qR has been solved (45) including an allosteric effect of zinc, which is required for binding HK and factor XII. It has been shown that HK, once bound to the cell surface, can be cleaved by adding kallikrein to release bradykinin (46) and one could activate the HK-prekallikrein complex, as bound to cells, even in the absence of factor XII. We purified a protein, heat shock protein-90 (HSP90) (47), which is secreted by endothelial cells and interacts with the complex of HK-prekallikrein (but not prekallikrein alone) to form a trimolecular complex which stoichiometrically converts the prekallikrein to kallikrein. The kallikrein thus formed can then activate factor XII under normal conditions. HSP 90 protein secretion can be induced by estrogen, interleukin 1, and TNFα, and also induces secretion of urokinase, which can bind to u-PAR and convert plasminogen to plasmin (48). Thus, the fibrinolytic cascade is also activated. Figure 7 is a depiction of the binding of factor XII and HK-prekallikrein to HUVEC as well as cell-surface reactions leading to bradykinin formation.

Apart from the aforementioned proteins, interactions of factor XII and HK with proteoglycans such as syndecan (49, 50) are also capable of modulating bradykinin formation (51), however, the effect of binding is inhibitory. The authors postulated that detachment from such binding is required for activation to proceed. Thus, we have two differing views of the mechanism(s) for factor XII and HK interaction with endothelial cells. These are not mutually exclusive, and it is likely that both are relevant but the contributions of one vs. the other and the circumstances that might affect HK and factor XII binding to protein complexes vs. proteoglycan are not known. We made one attempt to show evidence of a role for proteoglycan (sulfated mucopolysaccharide-protein complexes) in HK binding, and we approached it by enzymatically removing sulfates (which are thought to be essential for binding) and then checking the interaction of HK with the cell surface. It was unchanged (52). Thus, binding to the protein complex thesis was confirmed, and the proteoglycan contribution could not be quantitated. Nevertheless, a new form of HAE (discussed below), calls attention to a role for proteoglycan which, as implied above, may be inhibitory.

## HAE with normal C1INH

**Figure 8:**
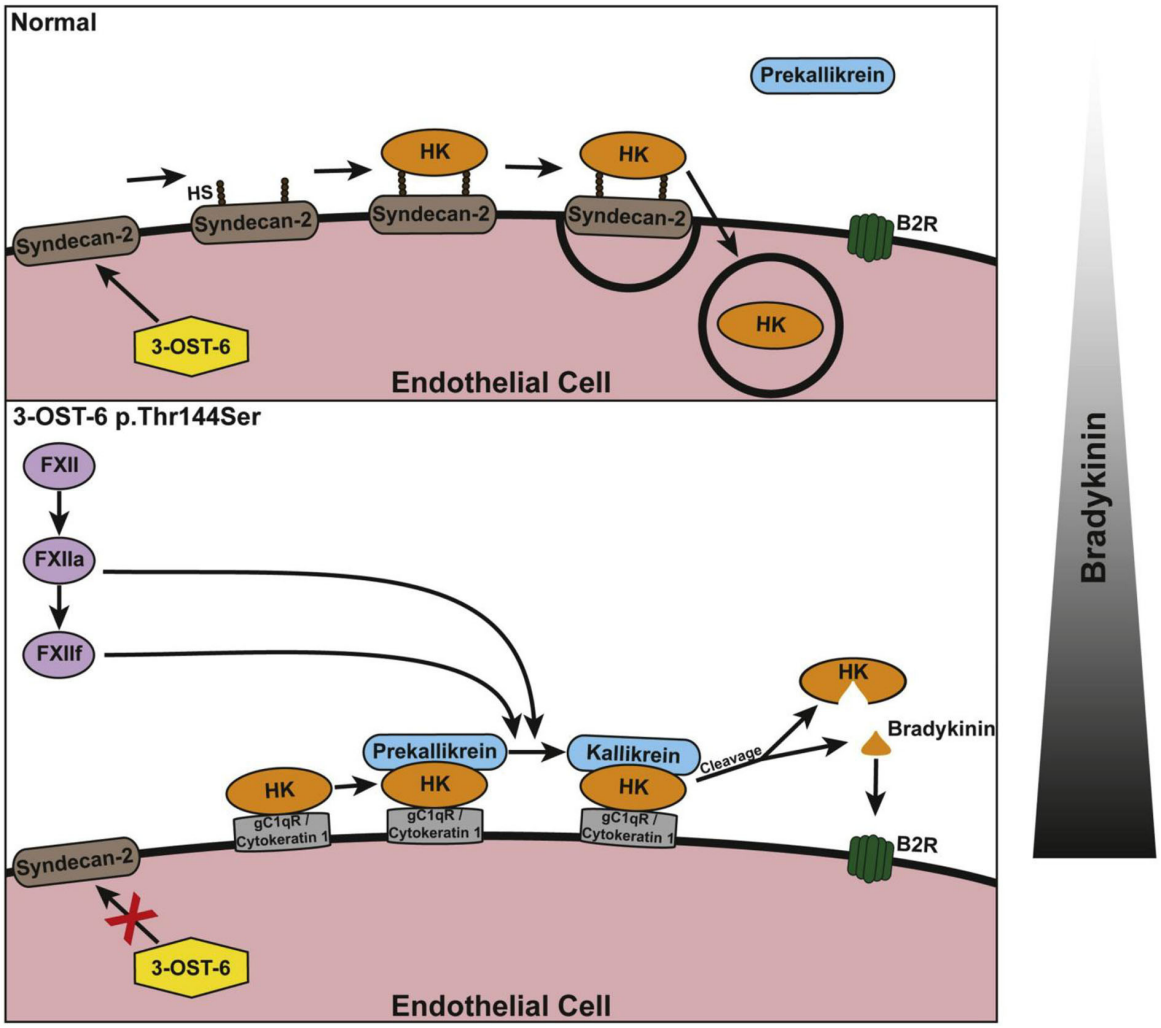
Schematic of the suggested involvement of 3-OST-6 in HK docking on the endothelial cell surface. The upper panel shows the normal (wild type) situation with HK being taken up *via* endocytosis due to interaction with heparan sulfate (HS)-containing proteoglycans. This prevents cleavage and bradykinin production. The lower panel shows the mutant situation: Because of incomplete HS modification, HK interacts with alternative interaction partners on the cell surface. This does not result in endocytosis and allows for HK cleavage and increased bradykinin production. The bar at the right indicates a shift in the balance of bradykinin production. Reproduced with permission: Bork et al. (60).

HAE with normal C1 INH has six forms thus far defined. Angiopoietin 1 and myoferlin mutations appear to be gain of function mutations that augment the effects of bradykinin upon endothelial cell receptors but do not appear to increase bradykinin formation (53, 54). The more common factor XII mutation (55) focuses on an augmented rate of factor XII activation (56, 57), by plasmin or thrombin, producing a truncated factor XII followed by cleavage by kallikrein (or plasmin) to activate it. A plasminogen mutation, however, appears to generate a mutant plasmin (glu^311^ plasmin) which directly cleaves kininogens, HK and LK, to produce bradykinin (58). Thus, factor XII and prekallikrein have been bypassed and a new, different enzyme produces bradykinin. There is one family with mutant HK, which in contrast to Williams plasma, has a mutation that leads to augmented bradykinin formation. That mechanism is thus far unknown (59). And finally, there is a family with a mutation in the heparin sulfate 3-0-sulfatransferase-6 gene that leads to HAE where there are two unproven assumptions: (A) that it leads to overproduction of bradykinin – this seems likely to be proven correct, and (B) the mutation diminishes sulfation of cell surface proteoglycan (60), but instead of decreasing bradykinin production, it leads to the opposite effect. With this information at hand, observations that shed light on this process include the ability of proteoglycans to assemble components (including HK) of the kinin-forming cascade (61), mediate endocytosis of HK with plasma prekallikrein to lysosomes (62), and then inhibit that endocytosis once bradykinin is released (63). A regulatory function for proteoglycan is thereby demonstrated. This is separate from the possible activation of factor XII by interaction with highly sulfated proteoglycan. It has been proposed that binding to proteoglycan (syndecan) is important at baseline and inhibits bradykinin formation and that with a defect in sulfation (as is depicted in Figure 8), the bimolecular protein complexes described above come into increased relevance (60) with activation of the cascade as a consequence. However, the protein complexes also appear to be significant at baseline (43). Thus, studies of the binding of HK and factor XII are needed that consider both binding to protein complexes and to proteoglycan at the surface of vascular endothelial cells in the same experiment. Nevertheless, the working hypothesis is that the mutation may eliminate an inhibitory effect of proteoglycan binding on bradykinin formation. These last two mutations (like the plasminogen mutation) may lead to a new understanding of the many routes by which *in vivo* bradykinin formation can occur.

## Concluding comments

There are data involving cleaved HK as an anti-adhesion molecule, and domain 5 of HK as an inhibitor of angiogenesis, and tumor formation, that I have not addressed (64, 65). The focus is on the multifaceted mechanisms by which HK simultaneously augments all the steps required for bradykinin formation and intrinsic coagulation and fibrinolysis, beyond being a substrate from which bradykinin is generated. These hopefully illuminate the ways in which this multi-domain protein is intimately involved in hereditary angioedema caused by C1 inhibitor deficiency, and beyond.

## Author contributions

All authors listed have made a substantial, direct, and intellectual contribution to the work and approved it for publication.

## Conflict of interest

The authors declare that the research was conducted in the absence of any commercial or financial relationships that could be construed as a potential conflict of interest.

## Publisher's note

All claims expressed in this article are solely those of the authors and do not necessarily represent those of their affiliated organizations, or those of the publisher, the editors and the reviewers. Any product that may be evaluated in this article, or claim that may be made by its manufacturer, is not guaranteed or endorsed by the publisher.
